# Pathogenic germline variants associated with different HER2 expression among breast cancer patients

**DOI:** 10.1007/s12672-026-05037-6

**Published:** 2026-04-28

**Authors:** Yongqi Ren, Li Cao, Chongyang Ren, Nanqiu Liu, Siqi Wang, Shuxuan Deng, Yan He, Cheukfai Li, Kai Li, Hsiaopei Mok, Lingzhu Wen, Yulei Wang, Xueying Zhang, Charles M. Balch, Jeffrey N. Weitzel, Guochun Zhang, Jiayan Wu, Ning Liao

**Affiliations:** 1 Department of Breast Surgery, Guangdong Provincial People’s Hospital, Guangdong Academy of Medical Sciences, Southern Medical University, 106 Zhongshan Er Road, Guangzhou, 510080 China; 2 Berry Oncology Corporation, Beijing, China; 3 Shantou University Medical College, Shantou, China; 4 Kanghui Biotech Corporation, Building 1, Yard 2, Guanzhuang Road, Chaoyang District, Beijing, 100101 China; 5 University of Texas MD Anderson Cancer Center, Houston, USA; 6 The University of Kansas Comprehensive Cancer Center, Kansas City, USA

**Keywords:** Breast cancer, Different HER2 expression status, Pathogenic germline variants, BRCA/FANC cluster

## Abstract

**Purpose:**

Germline mutations were evaluated in 530 Chinese breast cancer (BC) patients with different HER2 expression status plus 98 patients with atypical ductal hyperplasia or a breast cancer family history.

**Methods:**

DNA extracted from blood samples was analyzed with a next generation sequencing based multigene panel, with reporting of likely pathogenic and pathogenic variants (PV) of 102 cancer associated genes, and correlation between clinicopathologic characteristics and known BC-associated genes.

**Results:**

The 71 identified PVs were categorized into five distinct pathway clusters: BRCA/FANC, DDR, HRR, FANC, and Other. The distribution of PVs enriched within these clusters differed significantly between BC patients and unaffected individuals (*p* = 0.031). This difference was primarily driven by the BRCA/FANC, FANC, and DDR clusters, with enrichment percentages of 57.7% vs. 16.7% (BRCA/FANC), 7.0% vs. 25.0% (FANC), and 15.5% vs. 41.7% (DDR) in BC vs. unaffected groups, respectively. Notably, the three BC groups stratified by HER2 expression level exhibited distinct PV distributions. Both the HER2-low and HER2-zero BC groups showed significantly different distributions compared to unaffected individuals (*p* = 0.0132 and *p* = 0.0081, respectively). The BRCA/FANC cluster was the predominant pathway enriched in both HER2-low (59.6%) and HER2-zero (81.8%) groups. Furthermore, the PV distribution in the HER2-zero group was significantly different from that in the HER2-high group (*p* = 0.0028). In contrast, the distribution in the HER2-high group resembled that observed in non-BC individuals. These findings indicate that BC patients with different HER2 expression statuses harbor distinct germline PV signatures, which correlate with differential clinical outcomes following neoadjuvant systemic therapy.

**Discussion:**

Chinese breast cancer patients with different HER2 expression status demonstrated different pathogenic germline variant, which correlated with different clinical outcome after neoadjuvant therapy. Multi-gene genetic testing for pathogenic germline variants may be considered for breast cancer patients and high-risk individuals who could benefit from prevention and early detection programs.

**Supplementary Information:**

The online version contains supplementary material available at10.1007/s12672-026-05037-6.

## Introduction

Breast cancer (BC) remains the most frequently diagnosed malignancy among women worldwide [1]. According to the updated 2018 ASCO/College of American Pathologists guidelines for HER2 (human epidermal growth factor receptor 2) testing [2], breast cancers are now classified into three categories based on HER2 expression levels: HER2-positive (HER2-high), HER2-low (defined as immunohistochemistry 1+ or 2+ with negative in situ hybridization), and HER2-negative (HER2-zero). This classification has challenged the traditional paradigm that only HER2-high BC patients benefit from anti-HER2 targeted therapies [3], expanding treatment options—particularly antibody-drug conjugates (ADCs)—for HER2-low BC patients in various clinical scenarios [4–6].

Our previous research demonstrated that HER2-low BC exhibits distinct clinical and somatic mutational profiles compared to both HER2-zero and HER2-high subtypes [7]. Notably, over 10% of high-risk BC patients harbor pathogenic variants in cancer susceptibility genes [1]. This raises critical questions: Do germline pathogenic variants differ across HER2 status categories? And if so, how might these differences influence clinical outcomes?

Current population-based studies (primarily in Caucasian populations) have established several key findings regarding BC germline mutations: (1) Protein-truncating variants in *ATM*, *BRCA1*, *BRCA2*, *CHEK2* and *PALB2* increase BC risk [8], (2) *ATM* and *CHEK2* variants show stronger associations with ER-positive BC [8], (3) *BRCA1*/*2* and *PALB2* pathogenic variants confer high and moderate BC risk, respectively [9], (4) *BARD1*, *RAD51C* and *RAD51D* variants correlate with ER-negative and triple-negative BC [9], and (5) *ATM*, *CDH1* and *CHEK2* variants associate with ER-positive BC [9]. The BRIDGES project [10] further identified TP53 variants as having the strongest association with HER2-high BC, consistent with meta-analysis findings [11]. However, existing data remain limited—a study of 2538 Chinese BRCA1/2-negative BC patients found only 11 cases with TP53 germline variants [12], and no studies have reported germline mutation patterns stratified by HER2 expression levels.

This study analyzed pathogenic germline variants in a cohort of Chinese breast cancer patients stratified by HER2 expression status: HER2-zero (IHC 0), HER2-low (IHC 1+ or IHC 2+/FISH-negative), and HER2-high (IHC 3+ or IHC 2+/FISH-positive), along with unaffected controls. We hypothesized that distinct germline variant profiles may differentiate HER2-defined subtypes and potentially predict clinical responses to neoadjuvant therapy. Specifically, we aimed to address: (1) whether germline pathogenic variants differ significantly across HER2 status categories, and (2) the potential clinical implications of such variant distribution patterns on treatment outcomes.

## Methods

### Study design and patient enrollment

The study population included breast cancer patients with clinicopathological data including tumor class, HER2 expression status, histological grade, and hormone receptor (HR) status, and ancestrally matched non-breast cancer controls (Supplementary Fig. 1). In total, 530 Chinese woman with breast cancer, 98 women with atypical ductal hyperplasia and healthy participants (including 6 males) with family history of breast cancer were recruited between May 2021 to March 2023 at Guangdong Provincial People’s Hospital. Breast cancer patients were diagnosed with histologically confirm and have not received any systemic therapy. Basic information and pathological characteristics were collected from medical records. All clinical samples were conducted according to the ethical standards of the 1964 Helsinki declaration. The written informed consent for NGS-based genomic testing were signed and well-kept by all participants involved in this research.

### Immunohistochemical staining and standard HER2 testing

Available pathological data like HER2, ER and PR expression levels were detected by Immunohistochemical (IHC) staining and/or FISH. Molecular subtypes based on IHC and FISH were defined by according to the American Society of Clinical Oncology (ASCO)/College of American Pathologists (CAP) guidelines.

### Neoadjuvant therapy and treatment outcomes

A total of 115 breast cancer patients (21.7%) received neoadjuvant therapy with diverse treatment regimens. The therapeutic approaches encompassed:

- Endocrine therapy
- Targeted therapy
- HER2-directed therapy
- Immunotherapy
- Conventional chemotherapy
- Antibody-drug conjugates (ADCs)
- Epigenetic therapy
- Various combination therapies

### Genomic DNA extraction and sequencing

Blood samples were collected in EDTA tubes. Plasma was separated from blood samples by centrifugation (1500×*g*, 4 °C, 10 min) and re-centrifuged (16,000×*g*, 4 °C, 10 min) to remove cellular debris. Genomic DNA was extracted from 50 µL buffy coat by MagPure Tissue&Blood DNA LQ Kit (Magen, Guangzhou, China). Nucleic acid concentration was determined with the Qubit HS dsDNA kit (Invitrogen, Carlsbad, USA).

Germline variation profiling of the samples was performed using Hereditary Cancer Testing (Germline) (Berry Oncology, Fujian, China). The Hereditary Cancer Testing was designed to cover all coding regions and intron–exon boundaries of the 102 cancer susceptibility gene. NGS library was constructed using CS2.0 Tissue DNA Library Prep Kit (Berry Oncology, Fujian, China) according to manufacturer’s instructions. In brief, fragmented gDNA were end-repaired and A-tailed, followed by ligation with adapters containing unique molecular identifiers (UMI). Ligation products were purified and amplified by PCR. Amplified libraries were enriched for genes regions of interest by liquid hybridization using CS2.0 DNA Hybridization and Wash Kit (Berry Oncology, Fujian, China). The enriched library is quantified by real-time PCR and sequenced using NovaSeq 6000 (Illumina, San Diego, USA) in pair-end 150 bp mode for the average depth > 200X. Sequencing data was then analyzed by matched pipeline provided by Berry Oncology.

### Sequence data analysis

Bioinformatics pipeline developed by Berry Oncology was used to analyze the sequencing data. First, the sequencing reads were aligned to the reference human genome GRCh37 using bwa software (version 0.7.5a). Germline variations were called with GATK (version 3.8), Atlas2 (version 1.4.3), and VarDict (1). Annotations were defined using ANNOVAR (http://annovar.openbioinformatics.org/en/latest/) and TransVar [13]. Population allele frequencies were extracted from 1000 Genomes (http://www.1000genomes.org), dbSNP (http://www.ncbi.nlm.nih.gov/projects/SNP), and the Exome Variant Server (http://evs.gs.washington.edu/EVS).

### Identification and classification of germline variant

DNA from blood samples was subjected to targeted sequencing of 102 cancer susceptibility genes enriched in key DNA damage repair (DDR) pathways: the breast cancer-associated/Fanconi anemia (BRCA/FANC) [14], homologous recombination repair (HRR), Fanconi anemia (FANC), and other cancer-related pathways (Table S1). Variant curation was consistent with ACMG criteria [15], and statistical associations were determined between PVs in BC-associated genes and clinicopathogenic characteristics.

### Statistical analyses

Based on HER2 expression, patients were classified into three groups: HER2-zero, HER2-low, and HER2-high. For continuous variables, data were presented as mean and range; for categorical variables, data were presented as relative frequencies (percentage). Categorical variables were compared across groups using the chi-square test or Fisher’s exact test, as appropriate. Statistical analyses were performed using R version 4.2.2 (R Core Team). Two-tailed p-values < 0.05 were considered statistically significance.

To identify independent predictors of HER2 status, Firth’s logistic regression was applied to reduce small-sample bias. Each multivariable model included age, HR status, grade, stage, and one germline gene variant at a time. To account for multiple comparisons, we applied the Benjamini–Hochberg false discovery rate (FDR) correction as the primary method; Bonferroni-corrected results are also provided for reference. Associations with an FDR-adjusted p-value (q) < 0.05 were considered statistically significant (Table S2).

## Results

### Study population and clinicopathologic characteristics

**Fig. 1:**
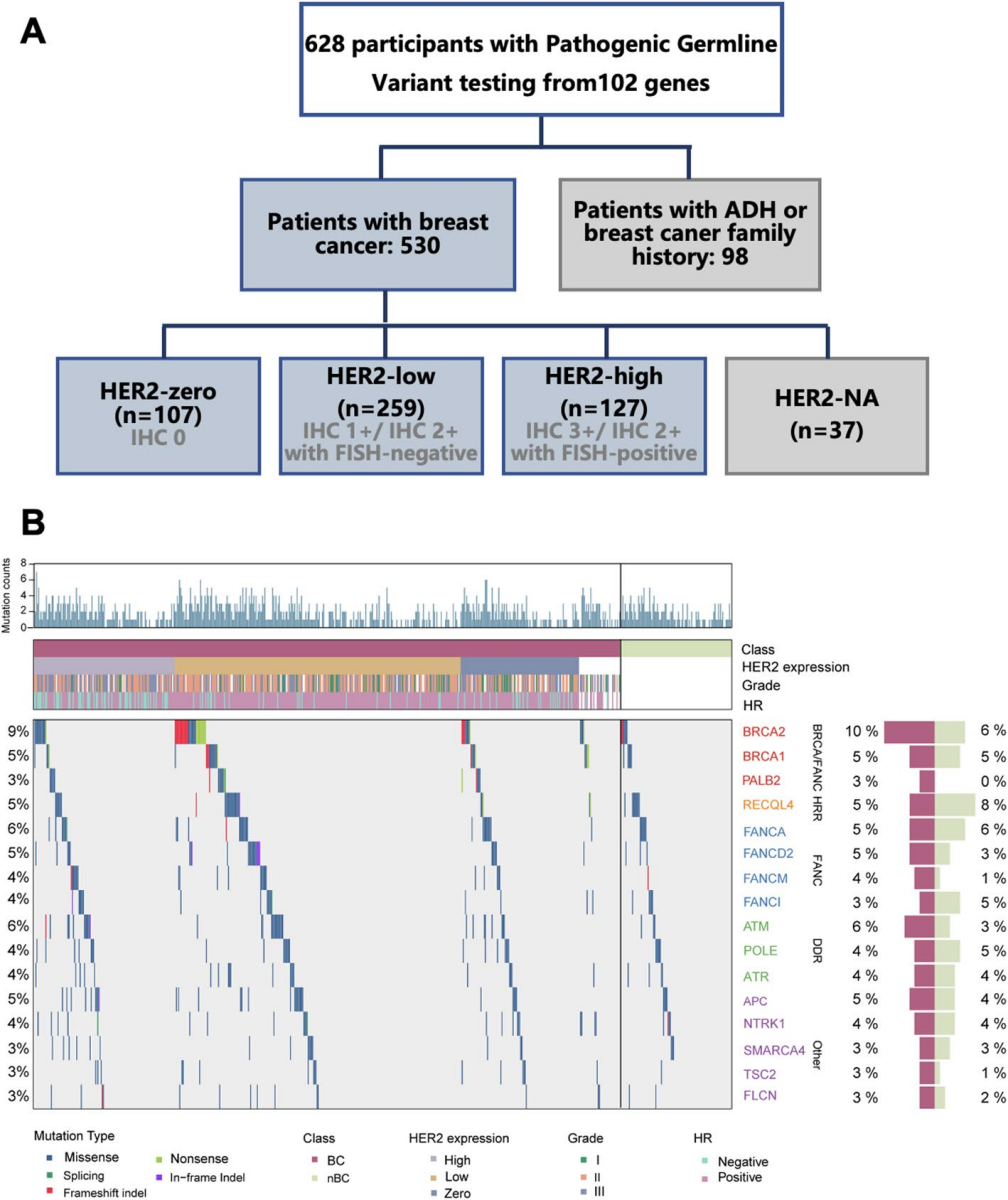
Study design and germline pathogenic variant (PVs) profile. **A** Diagram of the study design. **B** Oncoprint summarizing the mutated genes (up) and mutation type (down) per patient arranged according to each cluster as indicated at the top. Different colors at the top and bottom of the oncoprint represents other clinicopathological information of the patient including class, HER2 expression, grade, and hormone receptor (HR) status. And the different colors of gene names indicate different enriched pathways associated with the gene

Our study cohort comprised 530 breast cancer patients and 98 high-risk individuals (non-BC controls) with atypical ductal hyperplasia or family history of breast cancer (Fig. 1A). The baseline characteristics of the overall study population are summarized in Table 1. All breast cancer patients were female with a mean age of 48 years (range: 22–75 years). Notably, only 57 patients (10.8%) reported a family history of breast or ovarian cancer, and the majority were premenopausal at diagnosis.

**Table 1:** Clinical feature of the cohort

Clinical features	Breast cancer (BC)					Unaffected (non-BC)
	Total (*n* = 530); n (%)	IHC/FISH-based HER2 status subgroup; n (%)				Total (*n* = 98); n (%)
		HER2-zero (*n* = 107)	HER2-low (*n* = 259)	HER2-high (*n* = 127)		
Age (median [range], years)****	48 [22–75]	49 [29–68]	48 [22–75]	48 [23–59]		38[30–46]
Menopausal status						
Pre-menopause	316 (59.6%)	64 (59.8%)	158 (61.0%)	72 (56.7%)		–
Post-menopause	201 (37.9%)	41 (38.3%)	96 (37.1%)	52 (40.9%)		–
Unknown	13 (2.5%)	2 (1.9%)	5 (1.9%)	3 (2.4%)		–
Family history of breast or ovarian cancer****						
Yes	57 (10.8%)	12 (11.2%)	31 (12.0%)	13 (10.2%)		29(29.6%)
No	354 (66.8%)	77 (72.0%)	177 (68.3%)	87 (68.5%)		66(67.3%)
Unknown	129 (24.4%)	18 (16.8%)	51 (17.9%)	27 (21.3%)		3(3.1%)

* *p* < 0.05, ** *p* < 0.01, ***p < 0.001, ****p < 0.0001

Detailed clinicopathological features stratified by HER2 status are presented in Table 2. The cohort included patients across all pathological stages: 38.5% stage 0–I, 36.2% stage II, 16.0% stage III, and 4.6% stage IV. Tumor grade distribution was 10.2% grade I, 47.5% grade II, and 32.5% grade III.

**Table 2:** Clinicopathological characteristics of breast cancer cohort

Clinicopathological characteristics	Total (*n* = 530); n (%)	IHC/FISH-based HER2 status subgroup; *n* (%)		
		HER2-zero (*n* = 107)	HER2-low (*n* = 259)	HER2-high (*n* = 127)
Tumor (T) stage				
T1–T2	458 (86.4%)	96 (89.7%)	230 (88.8%)	113 (89.0%)
T3–T4	39 (7.4%)	8 (7.5%)	22 (8.5%)	9 (7.1%)
Unknown	33 (6.2%)	3 (2.8%)	7 (2.7%)	5 (3.9%)
Lymph node (N) stage				
N0–N1	427 (80.6%)	89 (83.2%)	209 (80.7%)	111 (87.4%)
N2–N3	85 (16.0%)	17 (15.9%)	49 (18.9%)	14 (11.0%)
Unknown	18 (3.4%)	1 (0.9%)	1 (0.4%)	2 (1.6%)
Pathological stage				
0–I	204 (38.5%)	43 (40.2%)	104 (40.2%)	44 (34.7%)
II	192 (36.2%)	38 (35.5%)	91 (35.1%)	60 (47.2%)
III	85 (16.0%)	18 (16.8%)	48 (18.5%)	16 (12.6%)
IV	24 (4.6%)	6 (5.6%)	13 (5.0%)	2 (1.6%)
NA	25 (4.7%)	2 (1.9%)	3 (1.2%)	5 (3.9%)
Grade**				
I	54 (10.2%)	14 (13.1%)	30 (11.6%)	5 (3.9%)
II	252 (47.5%)	49 (45.8%)	142 (54.8%)	53 (41.8%)
III	172 (32.5%)	33 (30.8%)	66 (25.5%)	62 (48.8%)
NA	52 (9.8%)	11 (10.3%)	21 (8.1%)	7 (5.5%)
Estrogen receptor (ER) status****				
Positive	395 (74.5%)	83 (77.6%)	230 (88.8%)	65 (51.2%)
Negative	118 (22.3%)	24 (22.4%)	29 (11.2%)	62 (48.8%)
NA	17 (3.2%)	0	0	0
Progesterone receptor (PR) status****				
Positive	346 (65.3%)	74 (69.2%)	215 (83.0%)	45 (35.4%)
Negative	163 (30.7%)	33 (30.8%)	44 (17.0%)	82 (64.6%)
NA	21 (4%)	0	0	0
Hormone receptor (HR) status****				
Positive	409 (77.2%)	86 (80.4%)	234 (90.3%)	74 (58.3%)
Negative	101 (19.0%)	21 (19.6%)	25 (9.7%)	53 (41.7%)
Unknown	20 (3.8%)	0	0	0
Neoadjuvant therapy*				
Yes	115 (21.7%)	18 (16.8%)	55 (21.2%)	41 (32.3%)
No	355 (67.0%)	81 (75.7%)	181 (69.9%)	73 (57.5%)
Unknown	60 (11.3%)	8 (%7.5)	23 (8.9%)	13 (10.2%)
Effect of neoadjuvant therapy**				
pCR	28 (24.3%)	4 (22.2%)	6 (10.9%)	18 (43.9%)
Non-pCR	56 (48.7%)	11 (61.1%)	31 (56.4%)	14 (34.2%)
Unknown	31 (27.0%)	3 (16.7%)	18 (32.7%)	9 (21.9%)

* *p* < 0.05, ** *p* < 0.01, ***p < 0.001, ****p < 0.0001

Based on HER2 immunohistochemistry and fluorescence in situ hybridization results, patients were classified into three groups (Fig. 1A): HER2-zero (IHC 0; *n* = 107, 20.2%), HER2-low (IHC 1+ or IHC 2+/FISH-negative; *n* = 259, 48.9%), HER2-high (IHC 3+ or IHC 2+/FISH-positive; *n* = 127, 24.0%). The patients in different HER2 groups had similar age (Supplementary Fig. 1A) and family history (Supplementary Fig. 1B). HER2-high tumors demonstrated distinct histological features, with significantly higher proportions of grade III tumors compared to both HER2-zero (51.3% vs. 33.0%, *p* = 2.8 × 10⁻³) and HER2-low groups (51.3% vs. 27.9%, *p* = 2.1 × 10⁻⁵; Supplementary Fig. 1C). Hormone receptor (HR) status analysis revealed that HER2-high tumors were less frequently HR-positive than both HER2-low (58.3% vs. 90.3%, *p* = 4.5 × 10⁻¹³) and HER2-zero tumors (58.3% vs. 80.4%, *p* = 5 × 10⁻⁴). Interestingly, HER2-low tumors showed the highest HR positivity rate, significantly exceeding that of HER2-zero tumors (90.3% vs. 80.4%, *p* = 0.014; Supplementary Fig. 1D).

### Germline pathogenic variant (PV) landscape in breast cancer patients

Our retrospective analysis identified 76 germline pathogenic variants (PVs) across 530 breast cancer patients and an ancestrally matched non-breast cancer control group. Overall analysis included 102 cancer susceptibility genes enriched in five key pathways: the breast cancer-associated/Fanconi anemia (BRCA/FANC) [14], homologous recombination repair (HRR), DNA damage repair (DDR), Fanconi anemia (FANC), and other cancer-related pathways (Table S1).

Figure 1B presents a comprehensive overview of mutated genes and variant types, organized alongside clinicopathological features including tumor class, HER2 expression status, histological grade, and hormone receptor (HR) status. *BRCA2/FANCS* from BRCA/FANC pathway category emerged as the most frequently mutated gene in both cohorts (10% mutation frequency), consistent with its established role as a tumor suppressor involved in key DNA damage repair, followed by *ATM* (6%), a critical kinase in the DDR pathway. Additional high-frequency alterations (all 5%) in breast cancer patients included: *BRCA1/FANCD1* from BRCA/FANC pathway, *RECQL4* from HRR pathway, *FANCA* and *FANCD2* from FANC pathway, as well as *APC* from other pathway category. We also performed multivariable logistic regression analyses for all 102 genes, adjusting for age, HR status, tumor grade, and stage. After applying false discovery rate (FDR) correction for multiple testing (Table S2), *CHEK2* mutations showed a nominal association with HER2-high tumors (OR = 21.9, *p* = 0.009), and RB1 (OR = 7.6, *p* = 0.022) and TSC1 (OR = 12.6, *p* = 0.039) also demonstrated nominal associations. But none of these associations retained statistical significance.

### Germline pathogenic variant heterogeneity across HER2 subgroups

**Fig. 2:**
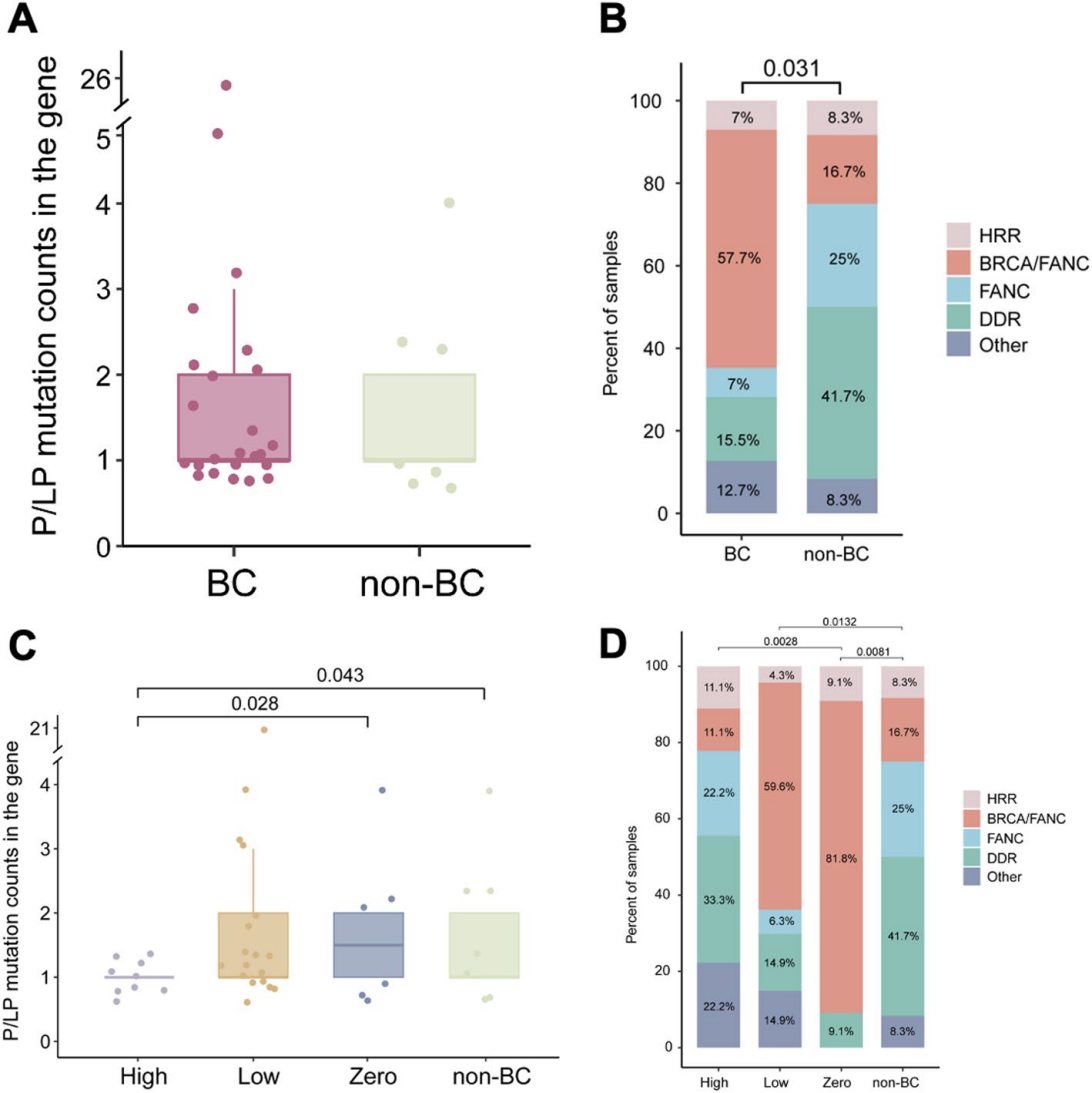
Germline pathogenic variant (PVs) distribution on the basis of pathogenic or likely pathogenic variant (P/LP) in the whole cohort. **A** Comparison of germline PVs counts between breast cancer (BC) and unaffected (non-BC) patients. **B** Comparison of germline PVs enriched in five pathway clusters between breast cancer and unaffected patients. **C** Comparison of germline PVs counts across HER2 groups (High, Low and Zero) and non-BC cohort. **D** Comparison of germline PVs enriched in five pathway clusters counts across HER2 subgroups (High, Low and Zero) and non-BC cohort

Current clinical genetic testing for breast cancer susceptibility primarily focuses on identifying pathogenic (P) and likely pathogenic (LP) variants. In our cohort, the variant count per individual of P/LP germline pathogenic variants (PVs) showed no significant difference between breast cancer patients and unaffected controls (Fig. 2A). However, when analyzing these variants by functional pathway clusters (BRCA/FANC, HRR, DDR, FANC, and others), we observed distinct distribution patterns between groups (Fig. 2B). Breast cancer patients exhibited significantly more BRCA/FANC-related gene mutations but fewer FANC and DDR related variants compared to unaffected individuals (*p* = 0.031).

Stratified analysis by HER2 status revealed further heterogeneity. The variant count per individual of PVs differed significantly (Fig. 2C) between HER2-high and HER2-zero groups (*p* = 0.028), as well as between HER2-high and non-BC groups (*p* = 0.043). Pathway cluster distributions showed significant variation (Fig. 2D) between HER2-high and HER2-zero (*p* = 0.0028), and between HER2-low and non-BC (*p* = 0.0132), and between HER2-zero and non-BC (*p* = 0.0081). Notably, HER2-zero and HER2-low patients carried substantially more BRCA/FANC and fewer FANC and DDR pathway variants, while HER2-high patients showed similar PV profiles to unaffected individuals (non-BC).

### Clinical outcomes stratified by HER2 status

Among the 530 breast cancer patients in our cohort, 115 underwent neoadjuvant therapy with various regimens, including endocrine therapy, targeted therapy, HER2-directed therapy, immunotherapy, chemotherapy, antibody-drug conjugates (ADCs), epigenetic therapy, or combination approaches (Supplementary Fig. 2). Of these, 84 patients had evaluable pathological response data.

**Fig. 3:**
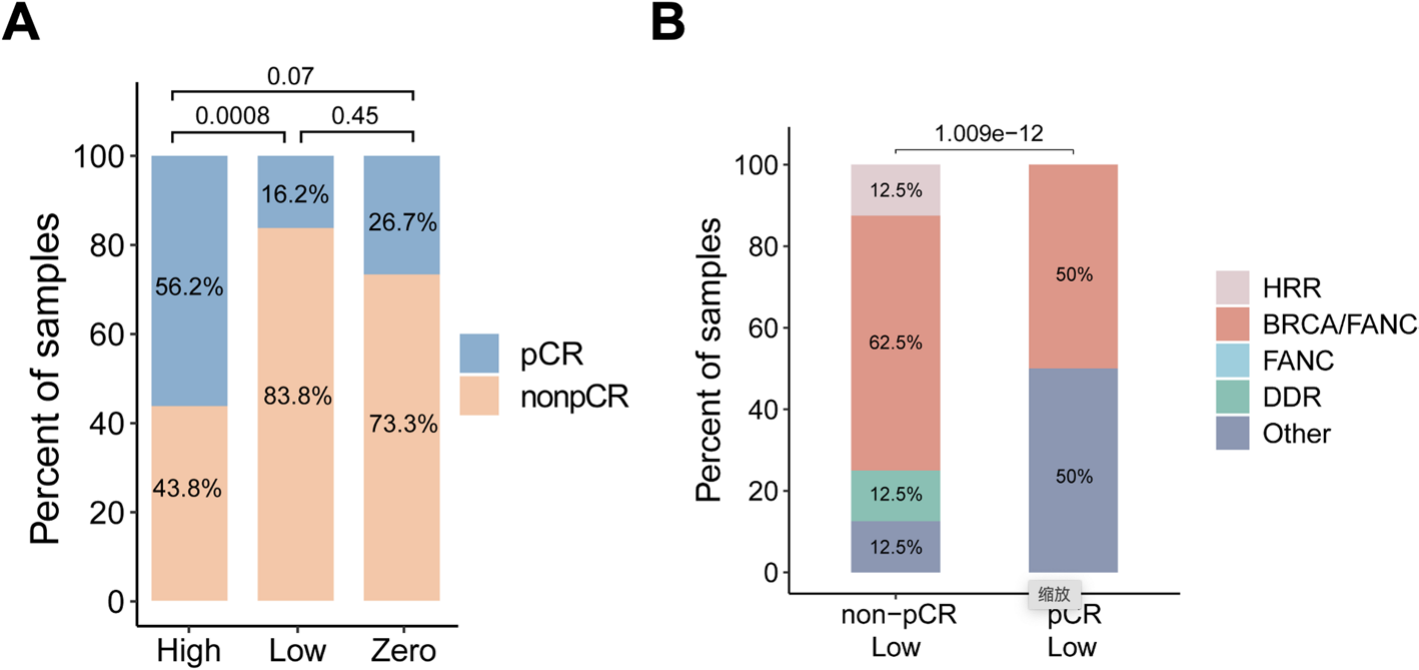
Clinical outcomes among HER2 subgroups (High, Low and Zero). **A** Comparison of pathological complete response (pCR) rates across HER2 subgroups (High, Low and Zero). **B** Comparison of germline PVs enriched in five pathway clusters between non-pCR and pCR patients in HER2-low subgroup. Statistical significance was assessed using Fisher exact test based on frequency comparisons. The small sample size notwithstanding, the large effect size between groups contributed to the observed p-value

Key findings regarding treatment response included:

1. Pathological complete response (pCR) rates (Fig. 3A):
  HER2-high patients demonstrated significantly higher pCR rates compared to HER2-low patients (*p* = 0.0008).
  A trend toward higher pCR rates was observed in HER2-high versus HER2-zero patients (*p* = 0.07).
2. Germline pathogenic variant associations (Fig. 3B):
  Distinct pathogenic germline variant profiles were observed between pCR and non-pCR patients across HER2 subgroups.
  In the HER2-low cohort, significant differences emerged in pathway-specific variant distribution between responders and non-responders (*p* = 1.009e−12).
  Non-responders in the HER2-low group showed particular enrichment of pathogenic variants related to BRCA/FANC and HRR and DDR pathway category.

This analysis reveals important interactions between HER2 status, germline variant profiles, and therapeutic response, suggesting potential biomarkers for treatment stratification.

## Discussion

In this study, we performed next-generation sequencing (NGS)-based analysis to evaluate germline pathogenic variants (PVs) in 530 Chinese breast cancer (BC) patients stratified by HER2 expression status, along with 98 individuals exhibiting atypical ductal hyperplasia or a family history of breast cancer serving as a non-BC control group. The clinical relevance of HER2 expression status has gained increasing attention due to the differential therapeutic benefits observed with novel HER2-directed antibody-drug conjugates (ADCs) [5]. Our research team has extensively investigated molecular characteristics across HER2 expression subgroups in BC [7, 16–21]. Notably, we previously demonstrated that HER2-low BC tumors harbor somatic mutations predominantly affecting the PI3K-Akt signaling pathway—a molecular profile associated with significantly lower pathological complete response (pCR) rates following neoadjuvant therapy [7]. Previous studies on HER2-low cohorts have primarily focused either on survival outcomes [22, 23] or on the efficiency of neoadjuvant therapy [24, 25], with cohort sizes approaching 20,000 subjects. However, none of these investigations examined the association between pathogenic germline variants and HER2 expression levels. Only M. Bottosso et al. reported a multicenter study investigating HER2 status and response to neoadjuvant anti-HER2 therapy in 232 patients with breast cancer and Li-Fraumeni syndrome [26]. To our knowledge, the present study represents the largest cohort to systematically evaluate germline genetic alterations across HER2-high, HER2-low, and HER2-zero breast cancer subgroups, with the inclusion of a non-breast cancer control cohort for comparative analysis.

Germline genetic variation analysis and associated genetic testing have garnered increasing attention in recent years, with accumulating research evidence and public health data significantly advancing our understanding of cancer susceptibility genes [27]—particularly in breast cancer [9, 28]. However, the germline mutational landscape across different HER2 expression subtypes remains poorly characterized. This knowledge gap is clinically relevant given that HER2-low breast cancer, representing approximately 55% of all breast cancer cases, predominantly exhibits hormone receptor-positive status [7–9]. Our study provides crucial insights into the distinct germline mutational signatures among HER2 expression subtypes and their differential responses to neoadjuvant systemic therapy. Through comprehensive multi-gene panel testing coupled with our novel pathogenic variant classification system, we observed significant heterogeneity in germline profiles across HER2-defined subgroups. These findings suggest the potential value of broad-panel germline testing, as genetic heterogeneity and overlapping phenotypic features may warrant evaluation beyond single-gene analysis [29]. The relevance of these observations is supported by the 2024 ASCO-Society of Surgical Oncology guideline update, which recommends multi-gene germline testing for breast cancer patients and high-risk individuals with a supportive family history, including genes beyond *BRCA1/2* to include other high-penetrance cancer susceptibility genes. Our findings in this HER2-stratified breast cancer cohort, together with non-breast cancer controls, appear consistent with these updated clinical recommendations.

We propose a novel classification framework for pathogenic variant (PV) identification, categorizing mutations into five key pathway clusters: the breast cancer-associated/Fanconi anemia (BRCA/FANC), homologous recombination repair (HRR), DNA damage response (DDR), Fanconi anemia (FANC), and others. The HRR and DDR pathways are critical for maintaining genomic stability, and pathogenic variants in these pathways are well-established drivers of cancer initiation, progression, and treatment resistance [15, 29]. More recently, the FANC pathway has garnered increasing attention due to its multifaceted role in DNA repair and tumor suppression. This pathway coordinates the repair of interstrand crosslink (ICL) lesions by interfacing with multiple DNA repair mechanisms [30]. Beyond ICL repair, the FANC pathway contributes to genomic integrity by regulating cytokinesis, stabilizing replication forks, and functioning as a crucial component of the tumor suppressor network [31]. Notably, the most extensively studied breast cancer susceptibility genes, *BRCA1*/*FANCS* and *BRCA2*/*FANCD1*, are integral components of the FANC pathway [32]. Additionally, other FANC-associated genes, including *PALB2* (*FANCN*) and *BRIP1* (*FANCJ*), have been firmly established as breast cancer predisposition genes [33, 34]. Therefore, Renaudin and Rosselli had defined the Fanconi anemia/breast cancer-associated (FANC/BRCA) pathway as the key hub protein complex for the regulation of the spatiotemporal repair of DNA interstrand cross-links (ICLs) [14]. Collectively, the BRCA/FANC, HRR, DDR, and FANC pathways form an intricate collaborative network to safeguard genomic stability, particularly in response to DNA interstrand crosslinks and double-strand breaks.

Our findings reveal striking differences in germline mutational patterns between BC patients (stratified by HER2 status) and non-BC individuals. Pathway analysis demonstrated significantly distinct distributions of PVs across five key biological pathways among the study groups, with the exception of HER2-high versus non-BC comparisons. Both HER2-low and HER2-zero subgroups exhibited markedly different PV profiles compared to the non-BC cohort. Although these two BC groups showed divergent PV distributions across pathway clusters, they shared enrichment of the breast cancer-associated/Fanconi anemia (BRCA/FANC)-related PVs—a finding with particular clinical relevance. This observation aligns with prior Western population studies (CARRIERS and BRIDGES projects [8, 9], which found defects in *BRCA1/FANCS*, *BRCA2/FANCD1*, *ATM*, *BRIP1/FANCJ*, *CHEK2*, and *PALB2/FANCN* confer elevated cancer risk [8]. In our HER2-focused cohort, BRCA/FANC-related PVs (predominantly *BRCA2/FANCD1*, *BRCA1/FANCS* and *PALB2/FANCN* mutations) were significantly associated with breast cancer development. HER2-low BC patients demonstrating poor therapeutic response to neoadjuvant regimens exhibited significantly different PV distributions across five pathway clusters. This suggests that BRCA/FANC, HRR and DDR pathway alterations might influence treatment resistance mechanisms in HER2-low breast cancer, suggesting potential targets for therapeutic intervention. This study also identified notable differences in pathogenic germline variants between Chinese and Western populations. Notably, no *CDH1* variants were detected in our cohort, and *TP53* variants—which show strong association with HER2-high breast cancer in Western populations (BRIDGES project) [10]—were observed in only one HER2-high case. These findings align with previous reports from Chinese *BRCA1/2*-negative breast cancer cohorts [16], reinforcing population-specific genetic profiles in breast cancer susceptibility.

We acknowledge that this study is associated with several limitations. First, the cohort size for individual pathogenic variants was relatively small, potentially limiting statistical power for rare variant analysis. Second, the proportion of patients receiving neoadjuvant therapy was suboptimal in our enrollment. Third, while the non-breast cancer control group was numerically matched to each HER2 subgroup, it remained disproportionately small compared to the entire breast cancer cohort, introducing potential selection bias.

In conclusion, our findings demonstrate that: HER2-defined breast cancer subtypes exhibit distinct germline variant profiles that correlate with differential neoadjuvant therapy outcomes, and PVs in HER2-low and HER2-zero subgroups enriched in *BRCA*/*FANC* pathway cluster. Given the therapeutic implications, multi-gene panel testing for germline mutations may be considered for breast cancer patients, high-risk populations, and potentially the general female population. These findings contribute to the evolving paradigm of precision prevention and treatment in breast cancer management, highlighting the potential value of population-specific genetic profiling and expanded genetic screening strategies.

## Supplementary Information

Below is the link to the electronic supplementary material.

Supplementary Material 1. Supplementary Fig. 1. Clinicopathologic characteristics in three breast cancer patient groups with different HER2 expression level (marked as High, Low and Zero in X-axis) as well as the breast cancer patients with unknown HER2 status (marked as NA in X-axis). A. Age B. Family history C. Grade D. HR status. Supplementary Fig. 2. Neoadjuvant therapy distribution (randomized). Endo, Endocrine therapy; TT, Targeted Therapy; HER2-T, HER2 Therapy; IO, Immunotherapy; CT, Chemotherapy; ADC, Antibody drug conjugate therapy; Epi, Epigenetic therapy.

Supplementary Material 2. Table S1: Detailed list of BRCA/FANC, HRR, DDR, FANC and other pathway genes of 102 gene panel. Table S2: Multivariable Logistic Regression Analysis of Associations Between Germline Variants and HER2-High Status.

## Data Availability

The datasets generated and analysed during the current study are available in the Genome Sequence Archive for Human (GSA-Human) repository, under accession number HRA 015770.
