# Supplementary material for: Pathogenic germline variants associated with different HER2 expression among breast cancer patients
Source: Discov Oncol. 2026 Apr 28;17:888. doi: 10.1007/s12672-026-05037-6 (PMC13253898; doi:10.1007/s12672-026-05037-6)
Supplement: Supplementary file 1 — Supplementary Material 1. Supplementary Fig. 1. Clinicopathologic characteristics in three breast cancer patient groups with different HER2 expression level (marked as High, Low and Zero in X-axis) as well as the breast cancer patients with unknown HER2 status (marked as NA in X-axis). A. Age B. Family history C. Grade D. HR status. Supplementary Fig. 2. Neoadjuvant therapy distribution (randomized). Endo, Endocrine therapy; TT, Targeted Therapy; HER2-T, HER2 Therapy; IO, Immunotherapy; CT, Chemotherapy; ADC, Antibody drug conjugate therapy; Epi, Epigenetic therapy. [file 12672_2026_5037_MOESM1_ESM.pdf]

Supplementary Figure 1

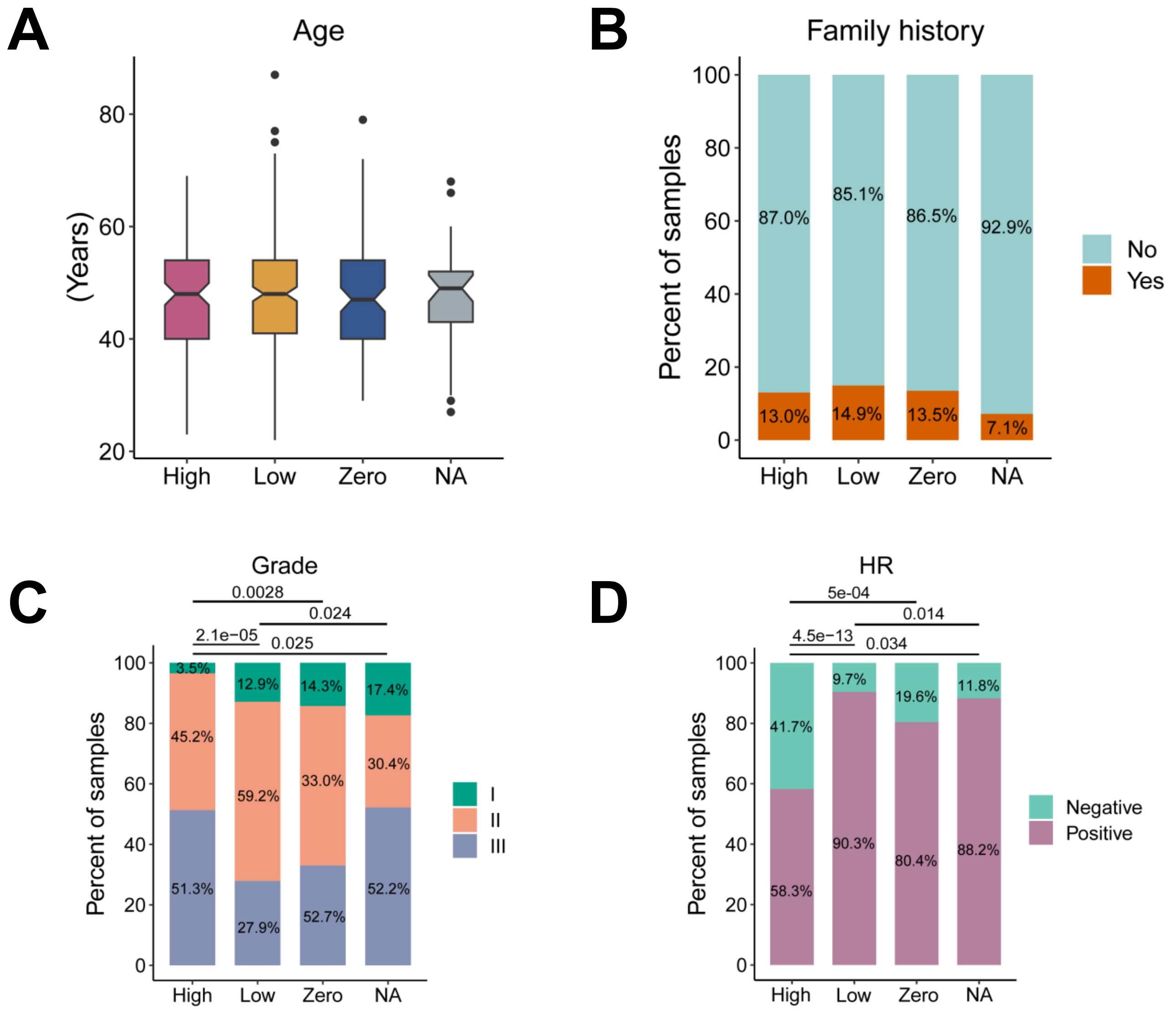

Supplementary Figure 1. Clinicopathologic characteristics in three breast cancer patients groups with different HER2 expression level (marked as High, Low and Zero in X-axis) as well as the breast cancer patients with unknown HER2 status (marked as NA in X-axis)

A. Age **B.** Family history **C**. Grade **D**. HR status

Supplementary Figure 2

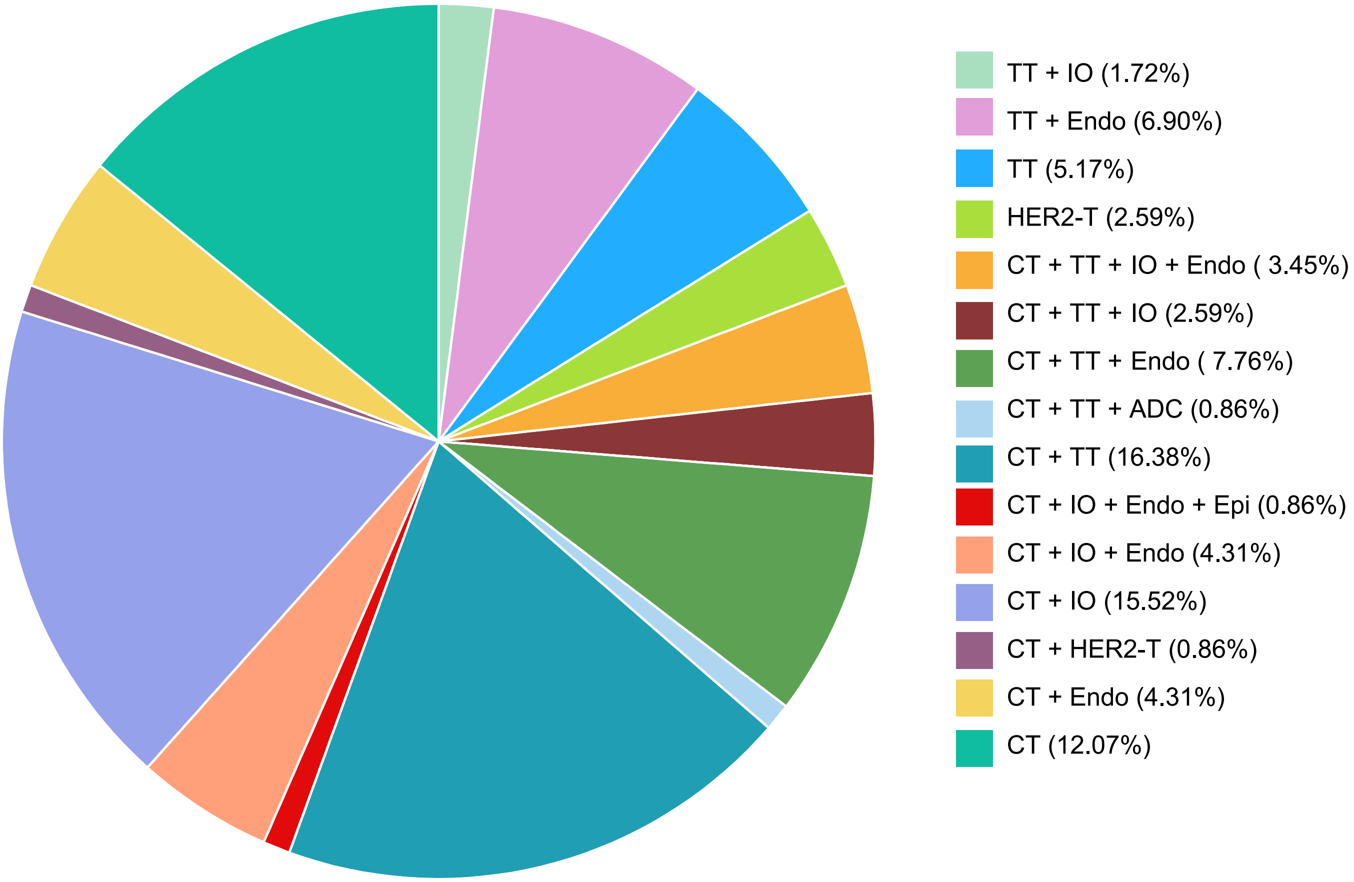

Supplementary Figure 2. Neoadjuvant therapy distribution (randomized). Endo, Endocrine therapy; TT, Targeted Therapy; HER2-T, HER2 Therapy; IO, Immunotherapy; CT, Chemotherapy; ADC, Antibody drug conjugate therapy; Epi, Epigenetic therapy.
